# Medications for opioid use disorders among incarcerated persons and those in the community supervision setting: exploration of implementation issues with key stakeholders

**DOI:** 10.1186/s13722-024-00528-9

**Published:** 2024-12-18

**Authors:** Augustine W. Kang, Amelia Bailey, Anthony Surace, Lynda Stein, Damaris Rohsenow, Rosemarie A. Martin

**Affiliations:** 1 Center for Alcohol and Addiction Studies, Brown University School of Public Health, Main St, Box G-121-5, Providence, RI USA; 2 Stanford University School of Medicine, Stanford, CA USA; 3 Department of Psychology, University of Rhode Island, Kingston, RI USA; 4 Department of Population and Quantitative Health Sciences, UMass Chan School of Medicine, Worcester, MA USA

**Keywords:** Opioid use disorder, Treatment provision, Criminal legal, Probation

## Abstract

**Introduction:**

Receipt of medications for opioid use disorder (MOUD) critically reduces opioid-related mortality during the post-incarceration period. Optimal provision of this care to individuals on community supervision (i.e., probation) requires an understanding of this unique and complex system at the local level.

**Methods:**

We conducted in-depth individual interviews with key treatment providers and probation staff (*n* = 10) involved with the provision of MOUD to individuals on community supervision in the Northeast. Interviews explored perspectives on the provision of MOUD and support services during the community supervision period. Thematic analysis was conducted to describe inductive and deductive codes, subcodes, and themes.

**Results:**

Stakeholders shared diverse attitudes about the benefits and drawbacks of MOUD utilization. The provision of MOUD during the community supervision period was perceived to be influenced by both treatment and probation organizational characteristics, including the structures and values of the agencies. As such, the specific context of the community supervision setting facilitated and impeded MOUD delivery. Persistent challenges to enhancing MOUD delivery to this population remain including widespread MOUD stigma, inter-agency communication issues, and structural barriers to healthcare (i.e., transportation, finances).

**Conclusions:**

There are opportunities to enhance access to evidence-based OUD treatment for persons on community supervision by engaging probation agencies and community treatment staff in systems change.

## Introduction

The opioid epidemic in the United States continues to escalate, with over 932,000 overdose deaths since 1999 [1]. In 2021, drug overdose deaths surpassed 100,000, with over 80,000 involving opioids [2]. Efforts to reduce prescription opioids have led to increased use of synthetic opioids like fentanyl [3, 4]. This crisis heavily impacts individuals in the criminal justice system, where substance use disorder rates are two to three times higher than the general population [5]. Released individuals face a heightened risk of overdose, particularly in the first two weeks [6]. 

Medications for opioid use disorder (MOUD), such as methadone, buprenorphine, and naltrexone, are effective for treatment [7–9]. Despite efforts to increase MOUD access in prisons, most programs are limited and not widely available to those under community supervision, with only 10% of the 50% needing treatment receiving it [10–12]. Expanding MOUD access in community supervision is crucial for managing the opioid crisis.

Community supervision (with the referral and treatment process beginning prior to release) provides a unique opportunity for early intervention, reducing recidivism through enhanced motivation for treatment [13]. However, individuals on probation or parole often lack proper evaluation and connections to treatment. Probation/parole agencies rarely provide MOUD due to lack of qualified staff and complexity in referrals [14, 15]. Systematic screening and referral processes are needed, requiring a blend of public safety and public health approaches [16]. Partnerships are essential to improve referral and assessment practices for individuals with OUD, addressing a significant gap in support.

Understanding community corrections staff attitudes and fostering organizational innovation is key for sustained change [17, 18] This study explores the role of correctional facilities and community treatment organizations in providing MOUD to individuals under community supervision through in-depth interviews with probation and treatment staff.

## Methods

This study was performed as part of the Justice Community Opioid Innovation Network (JCOIN) Providing Interventions for Enhancing Recovery during Supervision (PIERS) study [19]. It is a hybrid type 1 implementation effectiveness trial examining MOUD engagement in community supervision settings. Semi-structured interviews were conducted with probation/parole staff, leadership, and community health providers working with MOUD populations to explore their experiences, organizational influences, and perspectives on MOUD provision.

Participants were identified via purposive sampling with the following criteria: community health providers are defined as any treatment provider who has been involved in direct patient care for those on MOUD who are criminal justice-involved and have been employed in their role for at least three months; probation/parole staff are any probation/parole officer who has an active caseload, been employed in their role for at least 6 months. The interview guide queried three domains: (1) Organizational characteristics, which includes organizational climate in support of MOUD, (2) Knowledge and attitudes about MOUD, which includes their understanding of various types of MOUD (methadone, naltrexone (i.e., vivitrol), buprenorphine (i.e., suboxone)), benefits and drawbacks of MOUD, and implementation issues regarding MOUD in the community, (3) Perspectives towards populations on MOUD who are also under community supervision, including their experiences working with people under community supervision while on MOUD, and the barriers and facilitators to receiving MOUD for those under community supervision. The interview guide was cognitively tested with a member of the community before field implementation.

Interviews, conducted in 2021 via web conferencing, lasted 56 to 95 min, were recorded, and professionally transcribed. Data were analyzed iteratively, with a coding scheme derived from dominant themes. Open and axial coding was performed by two coders with a 97% inter-rater reliability. NVIVO Version 12 was used for analysis [20]. Informed consent was obtained from all participants prior to study administration. This study was approved by the Institutional Review Board at the University of North Carolina-Chapel Hill.

## Results

### Participant characteristics

Participants (*N* = 10) were on average 47.0±10.7 years old. See Table 1 for further demographics information.

**Table 1:** Participant Demographics

Variable (n=10)	Mean ± SD or N (%)
Age (Range: 32 - 66)	47.0 ± 10.7
Self-identified Gender	
Male	7 (70)
Female	3 (30)
Hispanic/Latino	0 (0)
Race	
White	10 (100)
Education	
Bachelor’s degree	3 (30)
Master’s degree and above	7 (70)
Job Title	
Probation/Parole Officer	5 (50)
Probation/Parole Supervisor/Leader	3 (30)
Treatment provider	2 (20)

**Fig. 1:**
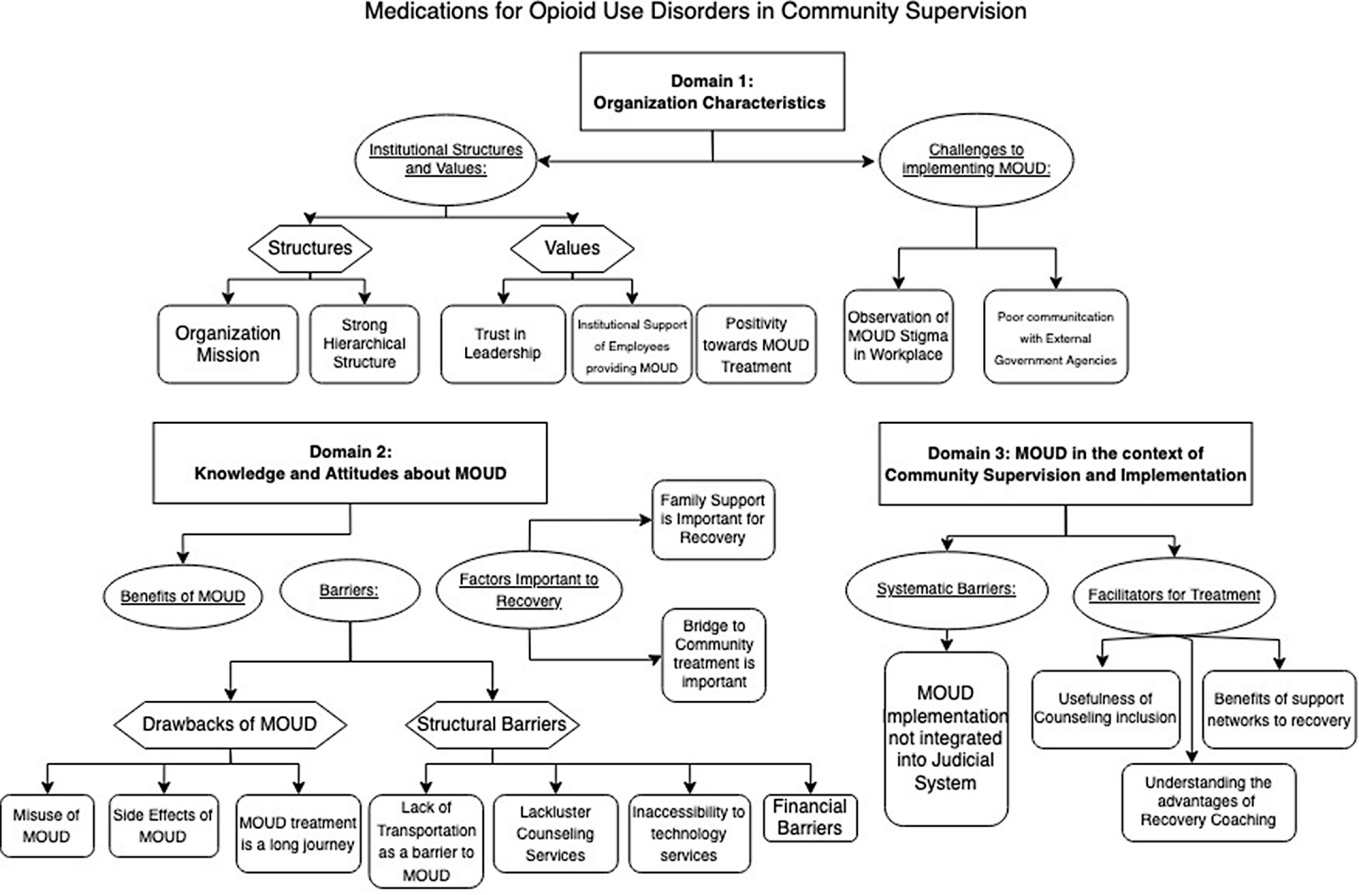
Illustration of Themes and Codes

Qualitative analyses revealed a plethora of factors related to MOUD treatment and usage among persons on probation. To help organize our findings, we report individual themes under broad domains around which they cluster (Fig. 1).

### Domain one: organization characteristics

The first domain of themes that emerged encompass how characteristics of providers’ organizations influenced MOUD treatment. Participants described how a variety of organizational-level features could both facilitate and hinder MOUD recovery trajectory.

### Sub-theme: institutional structures

#### Organization mission

Participants identified several organizational characteristics which impacted the use of MOUDs. For instance, the organization mission could facilitate utilization of MOUD for treating MOUD. As one participant described:

*I think*,* in my own words*,* and not reading from a script*,* the organization’s mission would be an individualized approach that best serves their needs. So*,* essentially a patient-centered approach to opiate use disorder. (*1009)

Other comments on their organizational mission include an emphasis on community safety and post-release success:

*I think the biggest priority is community safety. But the second biggest is offended change and trying to make sure that people succeed when they are either released from or get probation in the court system.* (1001)

#### Strong hierarchical structure

Participants also emphasized how there is a strong hierarchical structure in the department of corrections. For example, one participant said:

*It still tends to be a hierarchical situation because the department of corrections does have kind of a semi-military attitude.* (1005)

### Sub-theme: institutional values

#### Trust in Leadership

Participants describe how employees trust in their organizational leadership in their initiatives/policies.

*Yeah*,* I would say I trust our (leadership). You could call them supportive. They try to think of things help in outreach and stuff like that.* (1008)

And

*From the higher executive levels from the director’s office*,* and the executive team up they (are) always looking at a better way to do things and what works and making the necessary changes that we need to do that are appropriate for our department to succeed and do better.* (1004)

#### Institutional support of employees providing MOUD

Another important factor in MOUD usage described by participants was the institutional support of employees in providing MOUD. For example, multiple participants described how their superiors supported them and facilitated them providing care to clients on probation:

*I think my colleagues do feel supported (to provide MOUD) from the institutional standpoint. Because everyone is offered the same continuing education*,* access to conferences and resources as far as that goes. We meet as providers*,* discuss cases*,* discuss protocols in terms of how we plan on titrating doses…* (1009).

Such efforts by upper management appeared to be a deliberate effort to optimize care for those with MOUD. These anecdotes demonstrate that by creating an open and trusting environment for providers, they could “strategize” the best ways to treat those with MOUD.

As one supervisor described their approach:

*…at my site specifically*,* I try to create an environment where they’re free to talk about their concerns and share problems…How can we make this more productive for everybody? It’s just something that I can do to help as a program director.* (1008)

#### Positivity towards MOUD treatment

Participants described how their institutions utilized MOUD and are positive towards the use of MOUD when working with people who are incarcerated. For example:

*I (think) the addition of MOUD inside the facilities and using that as a model for when they’re released…has made a pretty big difference.* (1001)

### Sub-theme: challenges to implementing MOUD

Participants also described several factors which could hinder the utilization of MOUD at their organizations. Such factors included forces from both within and outside of their institutions.

#### Observation of MOUD Stigma in the workplace

Despite the overall positive attitudes towards MOUD, participants also suggested that some stigma towards MOUD also existed within their organizations. One participant described how:

*I think that I wish and hope that the community…I’d just like to see the stigma de-stigmatized. It’s heart-wrenching when they come in and they’re either like*,* “I’m not like the rest of your patients*,*” or the other day*,* we had somebody who was like*,* “Well*,* I don’t really want to come in every day because I might know somebody.”* (1008).

And

*I would say some probation officers do not agree with it. They feel like it’s just continued use. They think Methadone is just a poor substitute for the heroin or fentanyl use. They won’t agree to saying that someone completed treatment if they’re in that kind of program instead of a residential treatment program or completing at an outpatient level without using anything.* (1006)

#### Poor communication with external government agencies

Another barrier to MOUD utilization was potentially poor communication between agencies. Participants described how a lack of communication between organizations made it difficult to coordinate care. For example, through a lack of clear responsibilities between agencies:

*Here it’s all their own entities and battles and it’s not a lot of agencies working together as they should*,* in my opinion.* (1003)

### Domain two: knowledge and attitudes about MOUD

We examined participants’ knowledge, attitudes, and experiences on/with MOUD.

### Sub-theme: benefits of MOUD

Most participants described how MOUD is an effective treatment for OUD. This is best represented by the following:

*Well*,* I would imagine that if more people are successful on MOUD*,* you’re going to have less overdoses. You are going to have less people in need of medical care due to drug use or overdose. If somebody is taking care of themselves…hopefully*,* that’s going to trickle down to mean less ER visit.*. (1004)

Participants also describe that methadone, buprenorphine/naloxone, and injectable extended-release naltrexone are effective methods of MOUD. Unique quotes pertaining to each type of medication is captured below:

Methadone: *I think it probably works better for people that feel like they need to go every single day…people that are very or more routine-oriented. And I don’t know if actually going there and getting it every day helps them keep more cognizant of their use or if it’s a support to them*,* but I think people that probably are at highest risk…would be the benefit of Methadone.* (1007)

Suboxone: *Suboxone has less restrictions than methadone in regard to prescriptions and amount of medication they can hold on one time. So*,* if they’re working early hours or something like that*,* that conflicted with the clinic schedule…it’ll give them that flexibility that they’re looking for to be able to keep their work schedule.* (1008)

Vivitrol: *I have positive experiences in it working for clients. I feel like it’s less common as say Methadone* (1004);

and

*He’s been on the Vivitrol for I think two or three years successfully*,* no relapses. He comes in every four to six weeks*,* no issues. It’s a good treatment modality*,* but I think it really just needs to be selected for the right person*,* and the right person needs to be motivated for it.* (1009)

### Sub-theme: factors important to recovery

Participants discussed factors that were important to recovery. The common thread underlying these factors were: (1) that family support was key for recovery, and (2) bridge to community treatment was important:

*I think educating offenders’ families (is good)*,* it’s a really broad stakeholder thing*,* not everybody has to have the same level of understanding*,* but just enough to know that this is not a harmful thing… family support is really critical*. (1002)

And

*…From the beginning they talked about wraparound services. We have a strong discharge planning unit*,* that tries to bridge the gap between incarceration and community. We’re trying to use the continuation of services…so they have appointments when they (get) out.* (1003)

### Sub-theme: MOUD drawbacks, and barriers

Participants discussed the drawbacks of MOUD, as well as structural barriers to the implementation of MOUD.

#### Drawbacks of MOUD

In general, participants perceived that MOUD can be misused, their side effects, and that the recovery journey is long.

*When MAT[MOUD] first came around…when you hear Methadone*,* we had a lot of examples of offenders who would be sitting in front of you and they would be dozing off and their eyes would be shutting…they’re just nodding off.so it was not a good experience for us.* (1004)

Participants discussed how recovery is a long journey for clients on MOUD. Participants expressed concern that prolonged length time receiving MOUD may indicate that clients were misusing/dependent on MOUD:

*I remember her saying*,* she used to say to her doctor*,* I don’t want to be on this anymore*,* I want to be off this*,* but he would refuse …And then I can remember*,* she would say that she would wean herself off of it… She didn’t feel supported when she wanted to initially come off it…I want to say that it was a battle to get off it. This was not a six-month process. She was on it for a few years. And I know she wasn’t happy about that.* (1004)

#### Barriers to MOUD uptake

Participants described barriers to MOUD uptake, including the lack of reliable transportation that was potentially a deterrent to receiving MOUD, lackluster counseling services where MOUD counseling sessions were identified to be short and disengaging for patients, a lack of accessibility to technology services (that potentially can also be seen as a solution to transportation barriers), and financial barriers:

Transport: *The worst part about it*,* especially Methadone*,* was the fact that he had to go somewhere every morning and get it. So*,* for those people who didn’t have transportation or had to rely on somebody else for transportation*,* it was very difficult for some people to get to some of those clinics.* (1001)

Counseling: *I think more counseling or counselors at these programs are needed. I think they all have very high caseloads*,* so they don’t spend a lot of time with the offenders*,* and I don’t think they have as much as an impact on them…I feel like they also need the counseling part…* (1006).

Technology Accessibility: *Interviewer: In terms of access*,* transportation is a problem. Do you think that could be resolved by video conferencing technologies? Respondent: Yes. To an extent. With the DOC population*,* a lot of them don’t … I don’t even have phone numbers for … I have so many homeless people on this caseload and their phone numbers change all the time…technology could be a problem for some.* (1007)

Financial Barriers: *For methadone treatment specifically…(some patients) are just kind of in this weird bracket where*,* they make this a little too much to qualify for Medicaid*,* but they’re not really making enough to really support a monthly premium and then reaching those deductibles and things like that. Some of them… by the time they get the deductible*,* they could have paid full fee treatment the entire year and never reached the deductible.* (1008)

### Domain 3: MOUD in the context of community supervision and implementation

The final domain addresses the ways in which knowledge and attitudes towards community supervision influence the implementation and effectiveness of MOUD therapies.

Participants described how the current judicial system may not prioritize MOUD implementation as part of their processes:

*Most of the time*,* it seems like it (MOUD) is done on their (Clients’) own. It’s very rare that somebody is mandated to MOUD*^1^. *That’s one thing that I have in my experience*,* I can only think of one person that like part of their parole was to be on Methadone. There are a lot of clients who are on methadone*,* buprenorphine*,* naltrexone who are on probation or parole*,* but I have not necessarily seen that being mandated by the legal system. They will mandate outpatient counseling*,* mental health counseling and treatment but it’s very rare for MOUD.* (1010)

Just as how participants described how there were lackluster counseling services as an adjunct to MOUD, when participants were asked about how counseling can benefit populations in community supervision while on MOUD, they discussed the known benefits of counseling for addiction management:

*The benefits are as far as counseling…have always been really beneficial. We do mental health services and medication for mental health*,* and mental health counseling…majority of the clients are very happy by those services that I’ve provided.* (1010)

Participants also discussed the potential benefits of peer support specialists, such as the formation of a personal connection to someone with similar lived experience:

*A lot of them are recovered addicts… It’s not on so much of a professional level but it’s more on where the person’s at*,* more at their level. But they will reach out and keep reaching out and engage with the person and be there for the person.* (1007)

Social support was further highlighted to have a beneficial and critical role for the success among individuals on probation and MOUD.

*I think support from all levels*,* from the treatment agency*,* from your family*,* from your Probation Officer*,* from whatever counselor you’re seeing…from every single agency that you deal with*,* you need support.* (1001)

## Discussion

Our findings highlight opportunities to involve probation agencies in OUD treatment and provide direction for using community supervision as an approach to improve OUD treatment engagement and reduce overdose deaths among a population at-risk for health disparities. We identified that institutional structures are supportive of MOUD provision, though some challenges to implementation exist including MOUD stigma, inter-agency communication issues, structural barriers (e.g., transportation, lack of counselling services, inaccessibility to technology services, financial barriers). Importantly, we identified key formative information that will contribute to the future planned implementation of MOUD and recovery resource delivery among those under community supervision as part of JCOIN PIERS.

MOUD stigma persists as a barrier, particularly within the context of correctional systems. Individuals incarcerated for opioid-related offenses often encounter prejudices surrounding the use of MOUD as part of their treatment plan. This stigma is potentially rooted in misconceptions about the nature of addiction and the effectiveness of MOUD, as described by our participants. In the carceral setting, the lack of understanding and acceptance of MOUD contributes to inadequate access and provision of these evidence-based treatments. The lack of MOUD provision may lead to a downstream effect of relapse and recidivism [21]. Overcoming MOUD stigma within correctional settings requires education, destigmatization efforts, and policy changes to ensure that individuals have access to the most effective and humane addiction treatments, thereby fostering rehabilitation and reducing the risk of post-release substance use [22]. In the broader context of unintended consequences of stigma, some studies have showed those (e.g., cancer patients) with chronic pain experienced lower prescriptions rates, hence there is a need to untangle MOUD prescribing policy with stigma [23]. 

Though not traditionally involved in MOUD provision, probation and parole departments can play a crucial role in the successful implementation MOUD as part of comprehensive addiction care. As individuals under community supervision navigate the challenges of substance use disorders, probation and parole officers can serve as pivotal points of contact for assessing, supporting, and guiding them toward appropriate MOUD interventions. To do so however, probation and parole staff first need to be trained as organizational structures are currently not designed as service/treatment delivery systems. Overcoming these challenges requires a shift in perceptions and increased awareness of the effectiveness of MOUD to promote long-term recovery. The lack of a well-defined MOUD referral and treatment system in probation/parole settings results in a deficiency of essential support for individuals with OUD, heightening the likelihood of return to use. This gap emphasizes the need for informed partnerships to improve existing referral and assessment practices for individuals with OUD.

Barriers to MOUD implementation has been well-documented [24, 25]. Strategies to address barriers in the implementation of MOUD should recognize transportation barriers, with potential solutions being the provision of transportation assistance and leveraging telehealth services. The importance of community outreach and collaborative care models has demonstrable effects in MOUD engagement, and can potentially be used to address such barriers [26]. In this study, we highlighted specific barriers to address in the implementation of MOUD among populations under community supervision. Of interest, our study demonstrates organizational support and buy-in for the implementation of MOUD, which is characteristically different from some of the published literature [14]. This can potentially be explained by the fact that RIDOC’s unified jail and prison system was the first carceral facility in the US to provide access to all three types of FDA-approved MOUD to eligible individuals during incarceration, thus highlighting how this innovative setting may be distinct from other CL settings in the US. Importantly, staff buy-in is essential to ensuring mission alignment among key stakeholders involved in MOUD provision, and, in the absence of this buy-in, programs may face considerable challenges in facilitating MOUD referrals.

One of the focuses of our study was to explore the use of peer support specialists/recovery coaches for populations using MOUD while under community supervision. Peer coaches can play an important role in supporting those on MOUD – their unique lived experiences and can build a sense of trust and foster therapeutic alliance [27]. Peer coaches can help navigate barriers within the community supervision setting, such as the previously mentioned stigma and transportation challenges. Importantly, these coaches can provide a support system that can be a key ingredient in recovery [28]. Furthermore, in moments of crisis or relapse risk, peer recovery coaches can serve as frontline support.

A limitation to our study includes the specific structure of how probation and parole is organized within a single state in the Northeast, which limits the generalizability of the results. The sample size, while small, was able to yield rich discussion and data adequacy (given that we used purposive sampling to identify interview participants with significant organizational responsibility, impact, and/or experience) [29]. Future research might also consider including law enforcement officers’ and explore their understanding of MOUD as a treatment modality specific to this population. In addition, future studies might separately examine probation versus parole caseloads to determine if differences in MOUD treatment approach is needed (e.g., those involved in probation may not have been incarcerated, making probation officers the potential agent of change for this population).

This study highlights opportunities to involve community corrections agencies in OUD treatment and provides direction for using community supervision as an approach to improve OUD treatment engagement and reduce overdose deaths among a vulnerable population.

## Data Availability

No datasets were generated or analysed during the current study.

## Footnotes

1 As a commentary to this participant quote, the authors recognize that there is not a consensus that MOUD should be mandated due to ethical considerations. Instead, there is a need to advocate for access and integration of MOUD as part of a comprehensive, patient-centered approach.

## Declarations

### Statement of adherence to preprint policy

NA.
